# Chondroblastic osteosarcoma mimicking periapical abscess

**DOI:** 10.1590/1678-7757-2016-0424

**Published:** 2017

**Authors:** Fernanda Paula YAMAMOTO-SILVA, Brunno Santos de Freitas SILVA, Aline Carvalho BATISTA, Elismauro Francisco de MENDONÇA, Décio dos Santos PINTO-JÚNIOR, Carlos ESTRELA

**Affiliations:** 1 Universidade Federal de Goiás, Departamento de Ciências Estomatológicas, Goiânia, GO, Brasil.; 2 Centro Universitário de Anápolis, Departamento de Medicina Oral, Anápolis, GO, Brasil.; 3 Universidade de São Paulo, Faculdade de Odontologia, Departmento de Estomatologia, São Paulo, SP, Brasil.

**Keywords:** Osteosarcoma, Periapical abscess, Apical periodontitis, Differential diagnosis, Endodontics

## Abstract

Lesions of non-endodontic origin may mimic periapical abscess. Osteosarcoma is a rare malignant lesion.

**Case report:**

The present report describes a case of chondroblastic osteosarcoma in the periapical region of teeth #29, #30, and #31 of an 18-year-old male. Clinical history showed self-reported discomfort in the right posterior gingiva for over a month. Physical examination showed a small expansion and redness of the right mandibular buccal and lingual cortical plates, but no signs of pain or inflammation were observed. All the teeth responded positively to pulp sensibility. Periapical and panoramic radiographs showed slight periapical radiolucency in the roots of teeth #29 and #30, clear periodontal ligament space widening, and evident loss of lamina dura. Incisional biopsy was performed, and based on microscopic findings the diagnosis of chondroblastic osteosarcoma was confirmed.

**Conclusions:**

Non-endodontic diseases associated with tooth root apex, such as chondroblastic osteosarcoma, should be included in differential diagnosis of jaw lesions that resemble periapical abscess.

## Introduction

A periapical radiolucency associated with a vital tooth constitutes a diagnostic challenge^2^. Periapical lesions can be of endodontic or non-endodontic origin. Therefore, periapical radiolucency associated with root apices showed by radiographic examinations may be or not a consequence of infection of the root canal system^4,12,13^, which may involve progressive changes in periapical structures with subsequent bone resorption^12^. Conventional radiographic images are frequently used to detect apical periodontitis. The diagnosis of a periapical radiolucency requires careful and correct management of information obtained from patient history, clinical examination, pulp vitality testing, and radiography analysis^2,4,13^. The establishment of diagnostic procedures, such as examination of signs and symptoms, as well as complementary examinations, is indispensable to obtain differential diagnosis. Lesions of non-endodontic origin may be associated with the periapical area of the tooth^3,5,8,16,18^.

Osteosarcoma is a rare malignant neoplasm (incidence of 0.7 *per* million)^20^ of mesenchymal origin characterized by the production of immature bone^13^. It represents 5% to 13% of total osteosarcomas^6,7,9,10,13-15^ and occurs in the maxilla and mandible with approximately equal frequency^13^.

In the present report we describe a case of chondroblastic osteosarcoma resembling a periapical abscess in an 18-year-old male.

## Case report

**Figure 1:**
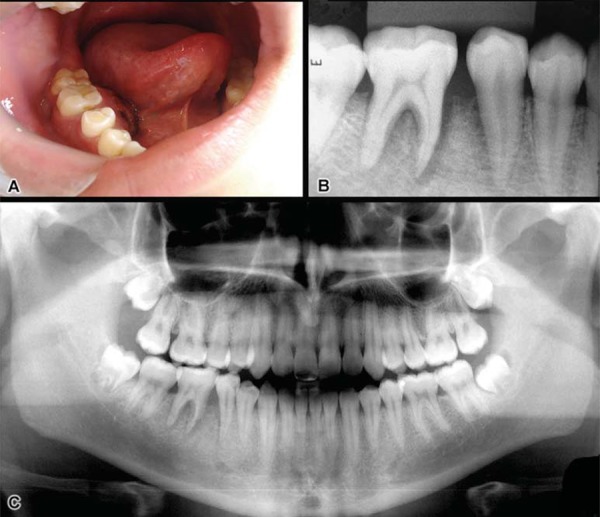
Clinical aspect (A) showing expansion of buccal and lingual cortical plates of the right mandible in the region of teeth #29, #30, and #31, with some redness and apparently intact overlying mucosa. Periapical and panoramic radiographs (B-C) showing slight apical radiolucency in the roots of teeth #29 and #30 with loss of lamina dura and periodontal ligament space widening

An 18-year-old male patient was referred to the dental clinic of the Federal University of Goiás (Goiânia, GO, Brazil) with a chief complaint of a “discomfort on the right posterior gingiva” for over a month. At physical examination, a small expansion and redness were found in the buccal and lingual cortical plates of the right mandible in the region of teeth #29, #30, and #31, but no signs of pain or inflammation were observed (Figure 1A). The overlying mucosa appeared intact.

Periapical and panoramic radiographs showed periapical radiolucency ranging from 4 to 5 mm in the mesial and distal roots of teeth #29 and #30, a slight rarefaction of the inter-dental alveolar bone, a clear periodontal ligament space widening, and an evident loss of lamina dura (Figure 1 B-C).

The patient reported no history of dental trauma. Neither cracks on the crowns of teeth #29 and #30, particularly on the mesial and distal marginal ridges, nor previous restorative treatments, were found. Pulp vitality testing using tetrafluoroethane spray (Endo-Ice; Hygenic Corp, Akron, OH) confirmed positive response in all teeth associated with radiolucent lesions, and therefore root canals were not treated.

**Figure 2:**
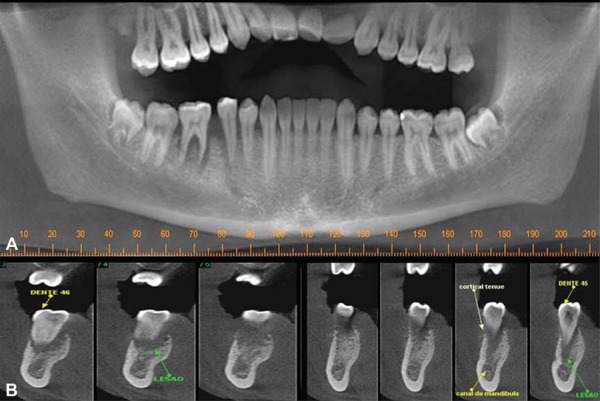
Cone beam computed tomography (A–B) showing a well-defined osteolytic lesion associated with the roots of teeth #29 and #30. Cross-sectional image (B) allowed observing alterations in the trabecular bone

**Figure 3:**
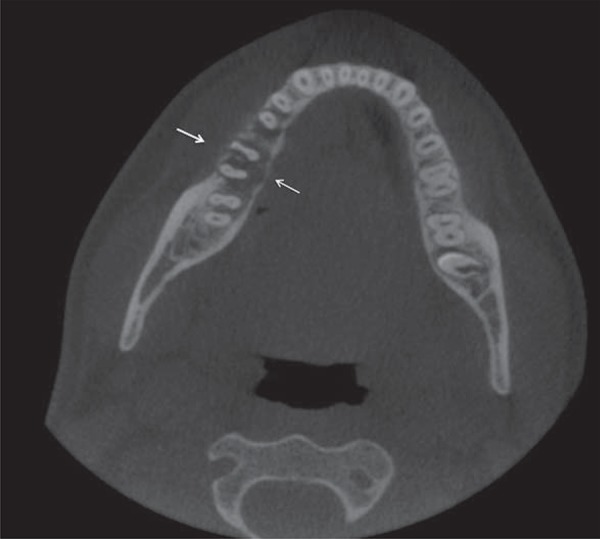
Axial plane cone beam computed tomography showing a thinning of the buccal and lingual cortical plates (arrows).

Cone beam computed tomography (CBCT) images were acquired. The imaging examinations showed undefined periapical osteolytic lesion, represented by a hypodense lesion with hyperdense areas inside, and thinning of the lingual cortical plate, associated with the roots of teeth #29 and #30. Alterations in trabecular bone were also seen (Figures 2 and 3).

**Figure 4:**
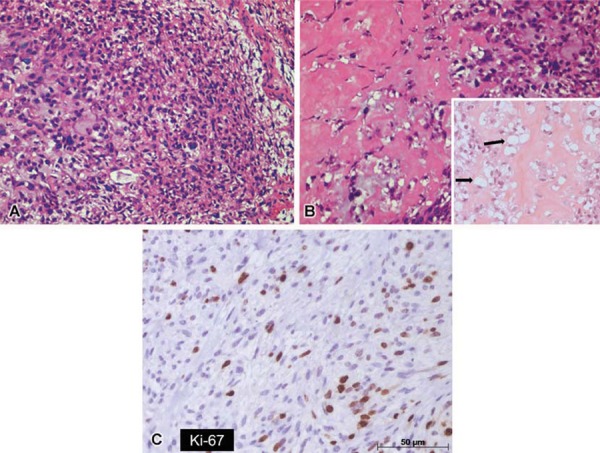
Histopathologic examination demonstrated round to spindle-shaped cells with cellular pleomorphism (A) and osteoid production (B). A focal area presenting proliferation of atypical chondroblastic cells was observed (arrows) (hematoxylin-eosin, original magnification 100X). Immunohistochemical reaction with Ki-67 (C) marker showed evident cellular activity in the specimen

Based on location of the lesion, its radiographic findings and the fact that periapical inflammatory lesions are the most frequent injuries in the affected region, the initial diagnosis included periapical abscess. However, given that the lesion had no relationship with pulp necrosis associated with the previous mentioned clinical and radiographic characteristics, intraosseous malignancies were considered, and an incisional biopsy was carried out. Microscopic evaluation showed a proliferation of round to spindle-shaped cells, with occasional cellular pleomorphism and variable osteoid production (Figure 4 A–B). Additionally, a focal area presented proliferation of atypical chondroblastic cells. Immunohistochemical reaction with Ki-67 marker showed evident cellular activity in the specimen (Figure 4C). Based on microscopic findings, a final diagnosis of chondroblastic osteosarcoma was confirmed.

The patient returned to the dental clinic 10 days after the incisional biopsy presenting with a considerable enlargement of the region. At this time, the patient was referred to Araújo Jorge Cancer Hospital (Goiânia, GO, Brazil). The treatment consisted of hemimandibulectomy with wide surgical margins and adjuvant chemotherapy.

**Figure 5:**
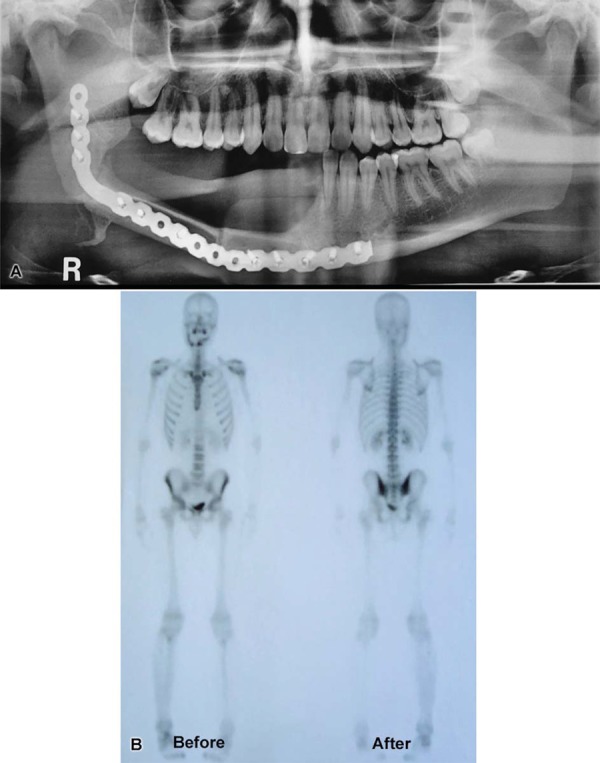
Panoramic radiograph (A) showing the reconstruction of the right mandible after surgical treatment. At the follow-up one year later bone scintigraphy (B) showed no signs of bone metastases

After surgical treatment, the patient underwent reconstruction of the right mandible with a fibular graft (Figure 5A). At the follow-up one year later, a bone scintigraphy was performed and no signs of bone metastases were seen (Figure 5B).

## Discussion

Osteosarcoma (osteogenic sarcoma) is a primary malignant tumor of the bone in which the neoplastic cells produce osteoid or bone matrix^20^. Excluding nonhematopoietic lesions, osteosarcomas are the most common primary malignant bone tumors^13^, which occur in the maxilla and mandible with approximately equal frequency^13,20^, predominantly in adolescents and young adults, between the ages of 10 and 20 years, and is slightly more common in males^20^.

An increase in volume in the affected area and pain are the most common symptoms of osteosarcoma^13^, and when it occurs in the maxilla, nasal obstruction may be present^13,17.^ The etiology of osteosarcoma are still unknown, however it has been associated with preexisting conditions, including prior radiation, fibrous dysplasia, Paget’s disease, and chronic osteomyelitis^19,20,21^.

In the case reported here, clinical findings were in accordance with previous descriptions of osteosarcoma^6,9,10,13-15,17,19,21,22^, since the patient was an 18-year-old man who complained of pain, the lesion was in the posterior region of the mandible, and clinical examinations showed a swelling in the affected area.

The clinical features of the lesion associated with presence of pain, swelling, and radiographic image of increased periodontal space mimicked a clinical condition of periapical abscess. Thus, based on these aspects, the initial differential diagnosis included periapical abscess. However, the positive response to pulp vitality testing suggested absence of root canal infection, which led to the recommendation of complementary examinations such as CBCT and an incisional biopsy. Due to the presence of an ill-defined radiolucency and periodontal space widening related with a vital pulp, intraosseous malignancies were considered in the differential diagnosis.

Common radiographic findings of osteosarcoma usually range from dense sclerosis to a mixed sclerotic radiolucent lesion and to a completely radiolucent process. The limits are undefined, making it difficult to determine tumor size and extension. The classic sunburst appearance is caused by osteophytic bone production on the surface of the lesion, especially in occlusal radiographs. An early radiographic change consists of a widening of the periodontal ligament space caused by tumor infiltration^13^. Considering the difficulty to determine tumor extension using conventional radiography, in the case reported here CBCT was used to determine the degree of bone destruction caused by the tumor, as well as its location and extension. A hypodense lesion with hyperdense areas inside, thinning of the lingual cortical plate, and an undefined periapical osteolytic lesion associated with the roots of teeth #29 and #30 were found.

The macroscopic essential criterion to characterize an osteosarcoma is the direct production of osteoid by malignant mesenchymal cells^13^. In addition to osteoid formation, tumor cells may produce chondroid material and fibrous connective tissue. Depending on the relative amounts of osteoid, cartilage, or collagen fibers, this type of tumor can be subdivided in osteoblastic, chondroblastic, and fibroblastic^1,13^. Osteosarcomas of the jaws are generally better differentiated than the extragnathic ones, and they commonly exhibit chondroblastic differentiation, characterized by lobules of atypical-appearing chondrocytes in lacunae^20^.

In the present report, the microscopic analysis showed a proliferation of round to spindle-shaped cells, with occasional cellular pleomorphism and variable osteoid production, as well as a focal area presenting proliferation of atypical chondroblastic cells. Cellular activity was confirmed by immunohistochemical reaction with Ki-67 marker. The histopathological examination confirmed the diagnosis of chondroblastic osteosarcoma.

Takahama, et al.^22^ (2003) analyzed the clinic pathological features and immunohistochemical expression of p53, MDM2, CDK4, PCNA, and Ki67 proteins in 25 head and neck osteosarcomas. The immunohistochemical analysis displayed positivity in 88% of the cases for Ki-67. Paparella, et al.^15^ (2013) analyzed 74 cases of osteosarcoma of the jaws and found a predominant chondroblastic pattern, which leads to the conclusion that these lesions may be associated with a worse prognosis. Bennet, et al.^1^ (2001) conducted a 30-year retrospective review of osteosarcoma of the jaws and compared the clinical behavior of the tumors. Their goals were to assess how they differ from the reported characteristics of tumors of other sites and to report observations of clinical and diagnostic significance. They reported that most osteosarcomas had areas of chondroid formation in addition to neoplastic osteoid, the main complication was local recurrence, and metastasis was rare and occurred as a solitary process or in late stages of the disease. This was in contrast to lesions metastatic to the jaws, which were higher grade in appearance and had metastasized widely, early in the disease process. Primary osteosarcoma occurring in patients with a history of radiotherapy was typically more aggressive.

The recommended treatment of osteosarcoma has historically been the surgical resection of the lesion with safe margins associated with chemotherapy^11,13,20^. In the present report, the treatment consisted of a hemimandibulectomy with wide surgical margins and adjuvant chemotherapy, due to the highly malignant features of the tumor, with a worse prognosis and a high risk of local recurrence. The patient underwent reconstruction of the right mandible, and clinical and radiographic follow-up one year later confirmed tumor remission. Bone scintigraphy was conducted and showed no signs of bone metastases.

In summary, osteosarcomas could present similar features of some inflammatory periapical lesions, such as periapical abscess, since it also present pain, swelling and variable radiographic changes^3^.

Exceptional care should be paid to endodontic diagnoses based on clinical and radiographic findings. Since periapical lesions of non-endodontic origin may mimic periapical abscess and apical periodontitis, they should be considered before root canal treatment.

## Conclusions

Non-endodontic diseases associated with tooth root apex, such as chondroblastic osteosarcoma, should be included in the differential diagnosis of jaw lesions that resemble periapical abscess. Periapical lesions may be misdiagnosed at their early stages if malignant tumors are not suspected.
